# Moving towards Environmental Sustainability: Can Digital Economy Reduce Environmental Degradation in China?

**DOI:** 10.3390/ijerph192315540

**Published:** 2022-11-23

**Authors:** Shunbin Zhong, Huafu Shen, Ziheng Niu, Yang Yu, Lin Pan, Yaojun Fan, Atif Jahanger

**Affiliations:** 1 School of Business, Minnan Normal University, Zhangzhou 363000, China; 2 Academy of Strategies for Innovation and Development, Anhui University, Hefei 230039, China; 3 School of Economics, Hainan University, Haikou 570228, China; 4 College of Oceanic and Atmospheric Sciences, Ocean University of China, Qingdao 266100, China; 5 Chinese International College, Dhurakij Pundit University, Bangkok 10210, Thailand

**Keywords:** digital economy, environmental sustainability, carbon emission intensity, regional heterogeneity, mediating effect, spatial spillover effect

## Abstract

In the context of environmental sustainability and accelerated digital technology development, China attaches great importance to the prominent role of digital economy in addressing environmental degradation. Utilizing Chinese provincial panel data from 2011 to 2019, this study investigates whether the digital economy can improve China’s environmental sustainability proxy by reducing carbon emission intensity. Based on the fixed effects model, the findings reveal that the digital economy has a significant negative effect on carbon emission intensity and the conclusion remains robust after conducting several robustness checks. However, this impact shows regional heterogeneity, which is more effective in resource-based eastern regions and the Belt and Road provinces. Moreover, mediating effect analyses indicate that the transmission mechanisms are energy consumption structure, total factor energy productivity, and green technology innovation. Furthermore, the results based on the spatial Durbin model (SDM) demonstrate that digital economy development has a significant spatial spillover effect. Finally, on the basis of results analysis and discussion, policy recommendations are provided for achieving environmental sustainability.

## 1. Introduction

This paper aims to find out whether digital economy can pave the way towards China’s environmental sustainability in the digital era, and to identify the mechanisms that may assist in achieving this. Environmental sustainability is defined as “meeting the resources and services needs of current and future generations without adversely affecting the health of the ecosystems that provide them” [1]. Environmental sustainability requires the conservation and protection of natural resources and global ecosystems to support public health and well-being now and in the future [2]. It not only plays a crucial role in economic growth, but also has a profound impact on long-term public health [3,4]. In 2015, the United Nations adopted a document entitled “Transforming our World: The 2030 Agenda for Sustainable Development” at the World Summit on Environmental Sustainability. Achieving sustainable development has certainly become a priority in modern society. However, environmental sustainability is difficult to achieve without reducing the unawareness associated with environmental degradation. Even though environmental protection has made some progress, there is still a serious problem with deteriorating ecological systems, unsustainable resource use, and climate change. In particular, carbon dioxide emissions are one of the primary causes of environmental pollution. Lessening carbon dioxide emissions can contribute to the achievement of the environmental sustainable development goals [5]. Thus, how to achieve carbon emissions reduction as a means of environmental sustainability has become an important issue under the current targets of carbon peak and carbon neutrality.

Digital economy refers to the increasing application and integration of digital technologies throughout the economy and society [6]. The advancement of digital technologies and their integration with the energy and environmental fields—such as energy Internet, smart energy grids, intelligent production lines, and energy-saving appliances—have presented tremendous changes in production and consumption activities. Digitalization through the development of information and communication technology (ICT) has been regarded as one of the most promising factors to reduce energy consumption and achieve sustainable development in the 21st century [7,8,9]. However, the impact of ICT on environmental sustainability remains controversial; some studies also argue that the application of ICT devices, such as big data centers and bitcoin blockchain operation, have increased energy consumption and may exert a negative impact on environmental sustainability [10,11]. ICT is only a single indicator of digitalization, and the digital economy involves multiple dimensions. It is therefore of great significance to construct an index capturing all facets of digitalization within an economy. In addition, little is known about the mechanisms through which digital economy affects environmental sustainability, especially for developing countries [12,13].

As the largest developing country and the world’s largest carbon dioxide emissions emitter, China is facing great challenges on the road towards its carbon neutrality goal. In the past, China has relied primarily on fossil fuels for its energy demand, especially coal consumption, which has accounted for approximately 70% of the main energy consumption [14]. The monotonous reliance on coal consumption and other unclean fuels has contributed to the surge in China’s carbon emissions and environmental degradation. At this stage, however, China’s digital economy has developed by leaps and bounds, accounting for 39.8% of China’s GDP with a scale of 45.5 trillion CNY in 2021 [15]. Digitalization has become the main engine of various economic and social fields. Given the fact that the digital economy plays an increasingly important role in China’s economic growth, will it be expected to minimize the country’s environmental degradation in terms of carbon emission intensity? If this effect exists, what are the influencing mechanisms underlying the effect of digital economy on carbon emission intensity? Moreover, will the digital economy’s impact on carbon emission intensity exhibit any regional and spatial heterogeneity? The answers to these questions provide theoretical and practical policy implications for the realization of environmental sustainability in China.

When compared with the existing literature, the main contributions of this study can be summarized as follows. First, this paper enriches the research on the internal relationship between digital economy and environmental sustainability and further extends earlier research findings. In contrast to previous research on environmental sustainability, it defines a unified framework of theoretical analysis and makes up for the field of digital economy and carbon neutrality. Second, this work builds a comprehensive digital economy index to systematically and comprehensively reflect China’s digital economy development based on the improved entropy weighted method, as well as the principal component analysis (PCA) method for a robustness check. The evaluation system for measuring digital economy contributes to a better understanding of digital economy’s environmental effects. Third, this research successfully identifies the channels of digital economy that affect carbon emission intensity and verifies its spatial spillover effect with the spatial Dubin model. Finally, this research provides policymakers with useful tools for reducing carbon dioxide emissions and enhances the government’s confidence in achieving environmental sustainability.

The remainder of the paper proceeds as follows: Section 2 provides a brief literature review of environmental sustainability and its correlation with digital economy; the theoretical analysis and research hypotheses are performed in Section 3; Section 4 explains the estimation methods and variable selection in this study; Section 5 presents the empirical results and a discussion; finally, the conclusions and related policy implications are provided in Section 6.

## 2. Literature Review

### 2.1. Environmental Sustainability and Its Influencing Factors

The existing literature concerning environmental sustainability primarily focuses on its measurement and influencing factors. In terms of environmental sustainability measurement, several indicators or sub-indicators have been considered in related research because it is a complex concept that encompasses environmental quality, social equality, and economic efficiency [16,17]. For example, Gómez-Limón et al. [18] construct a composite indicator for measuring environmental sustainability of olive farms in Spain based on a sustainability assessment of farming and environmental framework. Usubiaga-Liano and Ekins [19] provide a strong environmental sustainability index for 28 European countries by aggregating 21 indicators. Wang et al. [4] evaluate environmental sustainability based on the pressure-state-response (PSR) framework and the analytic hierarchy process method. Recently, attention has been given to the contribution of mitigating carbon dioxide emissions to the sustainability of the environment [20,21]. The higher levels of carbon dioxide emissions are the main challenge and threat to ecological welfare and environmental sustainability nowadays. Therefore, studies have shown that carbon dioxide emissions can be used as a proxy indicator for environmental sustainability [22,23,24].

In terms of factors influencing environmental sustainability, the environmental Kuznets curve has been discussed extensively by scholars [25,26,27], who generally believe that economic growth and environmental degradation exhibit an inverted-U relationship. In addition, other economic factors are also proven to have a significant impact on carbon dioxide emissions and environmental sustainability, including urbanization [28,29], financial development [30,31], foreign direct investment [32,33], trade [34,35], renewable energy consumption [36,37], and so on. In recent years, there has been a growing awareness of noneconomic factors on environmental sustainability, including environmental regulation [38,39], climate factors [40], research and development level [41], innovation factors [42,43], and technological progress [44,45].

### 2.2. The Nexus between Digital Economy and Environmental Sustainability

With the development of digital technology in recent years, the environmental impact of the digital economy has gradually attracted the attention of scholars around the world. The question of whether the digital economy improves ecological welfare is essential to achieving global environmental sustainability. Research on the digital economy–sustainable development nexus has primarily concentrated on the following three aspects, which can be clearly seen in Table 1.

The first stream of literature focuses on a single digital economy-related indicator to explore the digital economy–carbon dioxide emissions nexus. Salahuddin et al. [46], Lin and Zhou [47], and Wang et al. [48] find that there is a strong link between Internet development and carbon dioxide emissions. Moreover, mixed conclusions have been reached regarding e-commerce and carbon dioxide emissions because e-commerce can have both positive and negative environmental effects [49]. In addition, some recent research shows that digital finance has gradually become a vital factor affecting environmental quality through carbon dioxide emissions [24,62]. Furthermore, since information and communications technology (ICT) is closely intertwined with almost every sector in contemporary society, many studies have suggested that the adoption and application of ICT can have a significant impact on environmental performance [5,50,51].

The second stream of studies investigates the direct effect of digital economy on environmental quality indicators. Some studies have indicated that the advancement of digital technology directly leads to a rise in energy demand, which contributes to increased environmental degradation. Jiang et al. [11] analyzed the carbon emissions of China’s bitcoin blockchain and found that it would cause an incredible 130.50 million metric tons of carbon emissions in 2024. Asongu et al. [5] and Weili et al. [51] utilized the ICT sector as a representative of digitalization and found that ICT promotes the growth of carbon dioxide emissions in sub-Saharan Africa and the Belt and Road countries, respectively. Usman et al. [50] suggested that digitalization in terms of ICT penetration would cause serious environmental pollution in select higher-polluted Asian countries. However, other studies have remarked that digitalization is more conducive to promoting the improvement of environmental quality, which shows a reduction trend in energy consumption. Chiabai et al. [7], Bastida et al. [8], and Mansoor and Paul [9] provided evidence that digital technology can affect individual environmental protection behavior and household energy decision-making, thereby reducing energy consumption and achieving sustainable development. Chen [52] analyzed the digitalization in the form of internet penetration and proves that digitization is a blessing for decreasing carbon dioxide emissions. Furthermore, some relevant studies have found that ICT does not have a direct effect on carbon dioxide emissions in Tunisia or relatively high-income developing countries [53,54]. Therefore, there are conflicting arguments regarding the direct environmental impact of digital economy.

The third stream of research examines the effect of digital economy on environmental development from an indirect perspective. The digital economy is closely related to the environment and society, and several scholars have discussed this connection. First of all, Zhang et al. [55], Shahbaz et al. [56], and Xu et al. [57] have focused on the nexus of digital economy and energy structure. The results indicate that digitalization has notably increased corporate energy conservation and promoted energy transition. Second, the research of Canzian et al. [58] shows that digital technology can increase firms’ total factor productivity by 9.1%. Therefore, digital economy is an effective way to enhance China’s green total factor productivity [59,63]. Third, research has also shown that digital economy can promote green technology innovation by optimizing the industrial structure and stimulating innovation factor mobility [60,64]. Finally, there have also been studies that examine the nexus of digital economy and green financial investment [61], digital economy and human capital [65], and digital economy and the provision efficiency of public health institutions [66]. However, as a relatively new field, digital economy-related research has yet to be explored or addressed, including the relationship between digitalization and carbon emission intensity.

Furthermore, some scholars have pointed out that there is a spatial spillover effect between digital economy and environmental pollution. Shahnazi and Dehghan Shabani [67] use the dynamic spatial Durbin model to examine the spatial spillover effects between ICT and carbon dioxide emissions in Iran. The results confirm an inverted U-shaped relationship that considers the spatial spillover effects of ICT on carbon dioxide emissions. Zhou et al. [68] and Xu et al. [69] have noticed that digital economy has a substantial regional spatial spillover impact on haze pollution and environmental pollution. Hence, it is necessary to investigate the spatial spillover effects of the digital economy on carbon emission intensity in this study.

### 2.3. Literature Gaps

Although previous studies provide a solid foundation for our analysis, there are still some limitations and research gaps in this field. First, the intensive research concerning environmental sustainability primarily concentrates on its measurement and influencing factors. However, rare literature sheds light on the role of the digital economy due to the possibility that this field is relatively new. Second, preceding studies have mostly used a single indicator of digitalization, such as Internet development, ICT, digital finance, and so on. However, considering that digital economy involves multiple dimensions, such an approach may not be sufficient to capture all the facets of digitalization within an economy. Third, the current literature has mostly examined the direct effects of digital economy on environmental quality indicators, and has documented conflicting conclusions. Unfortunately, there is a lack of discussion on the possible internal mechanisms underlying the environmental effects of digital economy. Fourth, although some literature has discussed the effect of digital economy on energy structure, total factor productivity, and technology innovation, insufficient attention has been paid to the impact of the digital economy on carbon emission intensity. Moreover, there is still some uncertainty about the spatial spillover effect of regional digital economy on carbon emission intensity.

## 3. Theoretical Analysis and Hypotheses

**Figure 1:**
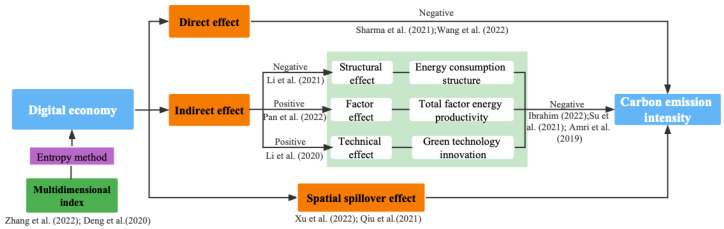
Mechanism analysis of digital economy on carbon emission intensity [20,42,53,70,71,72,73,74,75,76].

The impact mechanisms of digital economy on environmental sustainability can be decomposed into three paths: the direct effect path; the indirect effect path from the perspectives of energy consumption structure, total factor energy productivity, and green technology innovation; the spatial spillover effect path. Figure 1 illustrates the mechanisms through which a digital economy affects environmental sustainability in terms of carbon emission intensity.

### 3.1. The Direct Effect of Digital Economy on Environmental Sustainability

From the perspective of digital technologies, digital economy is more conducive to reducing carbon emissions than any other technology [70,77]. On the demand side, digital technologies are substituting physical goods with environmentally friendly products, such as emails, eBooks, online teleconferencing, e-commerce, and so on. Digital economy is an innovative way to encourage consumers to adopt eco-friendly products [9,78]. In addition, digital economy can effectively reduce final household electricity consumption by intervening in household energy consumption behavior [8]. Furthermore, digital economy has promoted the improvement of renewable energy technology and increased renewable energy consumption, thus supporting low-carbon sustainable development.

On the supply side, digital economy provides low-carbon technology support in the enterprise production process, thus greatly reducing carbon emissions. For instance, the digitalized and intelligent modifications of production equipment have suppressed environmental pollution in heavily polluting industries. With the application of digital technology, digital economy can facilitate information-based and dynamic environmental monitoring [71]. Thus, digital economy restricts the rent-seeking behavior of government officials and forces governments to pay more attention to environmental quality issues, thereby preventing and controlling environmental degradation. Consequently, based on this analysis, the following assumption is proposed:

**Hypothesis 1.**  *Digital economy can directly suppress China’s carbon emission intensity*.

### 3.2. The Indirect Effect of Digital Economy on Environmental Sustainability

Aside from its direct effect on carbon emission intensity, digital economy can also have potential indirect effects. These indirect effects can be divided into structural, factor, and technical effects, which are shown in Figure 1. First, structural effect refers to the fact that digital transformation can change energy-intensive heavy industries into low-polluting and knowledge-intensive industries [72]. Although China’s energy consumption has a rigid demand for coal, the proportion of high-energy consumption and high-polluting industries has decreased as a result of the impact of digital economy on industrial restructuring and upgrades. Digital economy is also favorable for enhancing industry 4.0 and increasing environmental carrying capacity to ameliorate the ecological environment [79]. Second, factor effect indicates that digital economy is an innovation driver for total factor productivity, which significantly facilitates high-efficiency and high-quality sustainable development. The innovation and application of digital industries and technologies has improved social economic productivity, especially for energy efficiency [73]. Digital economy can reduce the degree of energy resource mismatch, enhance energy-saving technological innovation, and reduce energy transaction costs. Thus, it eliminates the dependence of traditional economic development on fossil energy and promotes the reduction of carbon emissions by improving the total factor energy efficiency. Third, technical effect means that digital technologies can promote green technology innovation and thus lead to the reduction of carbon emissions. According to the green innovation theory, green innovation is the core driver of sustainable development by stimulating recycling technology, green product innovation, and green publicity [74]. In the production field, green technology innovation can improve the utilization ratio of unit resources and reduce carbon emissions via achieving cleaner production. Thus, this study proposes the second hypothesis as:

**Hypothesis 2.**  *Digital economy can indirectly inhibit China’s carbon emission intensity by optimizing the energy consumption structure, improving the total factor energy productivity, and promoting green technology innovation*.

### 3.3. The Spatial Spillover Effect of Digital Economy on Environmental Sustainability

The development of digital economy is characterized by external economic efficiency, cross-time and space, and openness. Through the transmission of excessive and high-speed information, digital economy is able to overcome the inherent time and space constraints and strengthen the inter-regional economic and production activities. Digital economy has accelerated technology and knowledge spillovers between regions, enhanced regional market access and industrial agglomeration, and generated a spillover effect in neighboring areas [69,80]. Regional economic activities have obvious spatial correlation, and the rise of digital technologies has promoted energy resource allocation, thus leading to a spatial spillover impact on carbon emissions. In contrast, Chinese local governments are interdependent through imitation and competition, and they will take competitive measures to develop the digital economy in order to maintain the carbon zero goals as well as economic benefits. In this process, spatial interdependence of digital economy development is evident among geographically interconnected Chinese provinces. Hence, this will also have a spatial spillover effect on carbon emissions performance. According to the above analysis, the third hypothesis can be proposed as follows:

**Hypothesis 3.**  *The impact of digital economy on carbon emission intensity has a spatial spillover effect*.

## 4. Methodology and Data

### 4.1. Models

The main purpose of this study is to investigate the impact of digital economy on environmental sustainability. As a means of capturing this effect, the econometric method is applied in this research. Referring to the settings of Salahuddin et al. [46], Wang et al. [71], Zhang et al. [75], and Shobande and Ogbeifun [81], the panel fixed effects model is first constructed as follows:

(1) $$lnCEI_{it}=\alpha_{0}+\alpha_{1}lnDEI_{it}+\sum_{j=2}^{n}\alpha_{j}lnX_{it}+\gamma_{i}+\lambda_{t}+\varepsilon_{it}$$

where the subscripts i and t denote a province and year, respectively. ln represents a natural logarithmic form of selected variables. *CEI* is the dependent variable and refers to the carbon emission intensity. *DEI* is the core explanatory variable, which can be used to analyze the development level of the digital economy indicator in each province. In addition, vector X is a set of control variables that may affect carbon emission intensity. Finally, ε represents the error term. In this study, the coefficient $\alpha_{1}$ is the key parameter identifying the influence magnitude of *DEI* on *CEI*.

In the next step, the mediating effect model is applied to evaluate the underlying mechanisms of how digital economy affects environmental sustainability. The literature shows the commonality of utilizing a mediating effect model when conducting mechanism analysis, as seen in the studies of Alesina and Zhuravskaya [82], Yang et al. [83], and Zhang et al. [75]. In particular, this article constructs the following two equations to represent the mediating effect model:

(2) $$lnM_{it}=\beta_{0}+\beta_{1}lnDEI_{it}+\sum_{j=2}^{n}\beta_{j}lnX_{it}+\gamma_{i}+\lambda_{t}+\varepsilon_{it}$$

(3) $$lnCEI_{it}=\varphi_{0}+\varphi_{1}lnDEI_{it}+\varphi_{2}lnM_{it}+\sum_{j=3}^{n}\varphi_{j}X_{it}+\gamma_{i}+\lambda_{t}+\varepsilon_{it}$$

where *M* is the mediating variable. In the mechanism analysis, the coefficients of interest are $\beta_{1}$, $\varphi_{1}$, and $\varphi_{2}$. When these three coefficients are all significant, it implies that the transmission mechanism exists. In particular, when the absolute value and significance of the coefficient $\varphi_{1}$ in model (3) are significantly lower than the coefficient $\alpha_{1}$ in model (1), it indicates that the variable lnM can play a mediating role in the effect of *DEI* on *CEI*.

Finally, due to the possibility of spatial autocorrelation in carbon dioxide emissions and the cross-regional cooperation in digital economy, this study further adopts the spatial panel model to explore the spatial spillover effect of *DEI* on *CEI*. The most-used spatial econometric models have been the spatial autoregression model (SAM), spatial error model (SEM), and spatial Dubin model (SDM) [84,85]. Compared with the SAM and SEM, the SDM has a relative advantage by considering the spatial hysteresis of both the dependent variable and core explanatory variable. Thus, the SDM plays an important role in practical applications, and it can be determined by the following equation:

(4) $$lnCEI_{it}=\delta_{0}+\rho \sum_{k=1}^{n}w_{ik}lnCEI_{it}+\delta_{1}lnDEI_{it}+\delta_{2}\sum_{k=1}^{n}w_{ik}lnDEI_{it}+\sum_{j=3}^{n}\delta_{j}X_{it}+\gamma_{i}+\lambda_{t}+\varepsilon_{it}$$

where ρ represents the spatial autoregression coefficient, indicating the spatial spillover effect of adjacent geospatial regions. w refers to the spatial weight matrix. Additionally,$\;\delta_{2}$ captures the elastic coefficients of spatial interaction terms for *DEI*.

### 4.2. Variable Selection

#### 4.2.1. Dependent Variable

Carbon emission intensity (*CEI*) is measured by the amount of regional carbon dioxide emissions per unit of GDP, which indicates the degree of damage to the environment [43]. In the domain of environmental sustainability, numerous scholars have discussed the strong link between environmental sustainability and carbon dioxide emissions [22,23,24]. Environmental degradation due to carbon dioxide emissions is one of the biggest concerns for human survival in the context of a new era. Reducing *CEI* has become the key to achieving the goal of carbon neutrality and environmental sustainability. Hence, this study takes *CEI* as the leading indicator of environmental sustainability.

Considering that China’s total carbon emissions are primarily caused by energy-related sources [23], this research assesses environmental sustainability from an energy consumption perspective. Specifically, there are seven types of energy selected for calculating carbon emissions, including natural gas, kerosene, gasoline, coke, diesel, coal, and fuel oil. In conjunction with the unified standard developed by the Intergovernmental Panel on Climate Change (IPCC) and relevant parameters from China’s energy research institute, Table 2 presents the carbon emission parameters of these energy consumptions.

Based on the above estimation of carbon emission parameters, the indicator of the *CEI* can be calculated by applying the following specific formula:

(5) $$CEI=\frac{1}{GDP}\sum_{i=1}^{7}CE_{i}=\frac{1}{GDP}\sum_{i=1}^{7}E_{i}\times CAC_{i}\times CAV_{i}\times COF_{i}\times \frac{44}{12}$$

where i refers to the energy type, *GDP* is the gross domestic product of each province, *CE* denotes the total carbon emissions of each province’s energy consumption, *E* is the total energy consumption of each province, *CAC* represents the carbon content of various energy, *CAV* refers to the average calorific value, and *COF* denotes the carbon oxidation factor.

#### 4.2.2. Core Explanatory Variable

Considering the fact that a single index cannot adequately capture the ecological impact of the digital economy, this study applies the improved entropy weight method to construct a digital economy indicator (*DEI*). In the field of environmental economic statistics research, the entropy method is a relatively scientific method of evaluation. Compared with the traditional entropy approach, the improved entropy weight method has at least two advantages for the study of regional digital economy [75,76]. Firstly, it can effectively address the problem that the weight of a single indicator of digital economy is too large or too small. Secondly, it can not only retain the information entropy characteristics of the digital economy, but also eliminate the influence of high discrete values on digital economy measurement. Therefore, the improved entropy weight method is well suited to calculate the regional digital economy development level in this study. This method can assign weights to each selected sub-indicator and characterize digital economy development to the greatest extent [86]. The higher the value, the more developed the digital economy is. Drawing on the practice of Zhang et al. [75] and Ma et al. [87], the indicator system of China’s digital economy development is established from four perspectives: digital industrialization, digital transaction, digital infrastructure, and digital literacy. Specifically, there are 13 sub-indicators selected to comprehensively and accurately evaluate China’s digital economy development. Table 3 shows the details and calculated weights of the indicator system.

At present, there are primarily two parallel paths to the digital economy’s interaction with the economic and environmental systems: digital production and digital application. In terms of digital production, the digital economy is accompanied by the digital industries and the transition of new businesses. Therefore, digital industrialization and digital transaction are taken into account as indicators of the digital economy. The indicators of total revenue of telecommunications and software per capita, as well as the ratio of practitioners in the information industry, are combined to reflect the digital industrialization. Moreover, digital transaction can be measured by indicators related to the digital financial inclusion, express delivery industry, and the tertiary industry [88,89]. In terms of digital application, the indicators of digital infrastructure and digital literacy have become a key driving force behind the development of the digital economy. In this study, key indicators of the construction of digital infrastructure are collected, including the penetration and diffusion of broadband Internet and mobile Internet, the long-distance optical cable density, the number of domain names, and the Internet access ports density. Finally, digital literacy includes indicators representing the level of education and education expenditure.

#### 4.2.3. Control Variables

Along with the existing studies, this study controls the following variables to minimize the potential error arising from missing variables:

Economic development (*Rgdp*): Regional real gross domestic product (GDP) is regarded as the indicator of reflecting the economic development stage. It is worth noting that the nominal GDP of each year was reduced based on 2011 GDP to eliminate the impact of price changes. The influence of economic development on carbon emissions has been confirmed in many studies, and it should be introduced into the control variables [34,90].

Urbanization level (*Urban*): The urbanization rate defines the number of people moving from the rural to the urban regions, which is the ratio of the urban population to the total population [29]. Global carbon emissions have been driven by the growing population, and rapid urbanization has tremendously increased energy consumption. Referring to the study of Zi et al. [28], approximately 84% of total commercial energy is consumed in China’s urbanized areas.

Foreign direct investment (*FDI*): Actual foreign direct investment as a percentage of GDP is selected as an indicator of measuring FDI development. FDI is a major contributor to carbon emissions, and there is a two-sided effect of FDI on carbon emissions performance in China [33]. FDI can not only promote but also inhibit the carbon emissions, which has been viewed as two opposing hypotheses: “pollution paradise” and “pollution halo” [32].

Trade structure (*Trade*): It is calculated by ratio of total exports and imports to the GDP [91]. Carbon emissions transfer globally along with trade openness due to the globalization of goods and services. The carbon intensity caused by trade openness has become more and more obvious in recent years [35].

Research and development level (*Rdl*): The research and development expenditure as a share of GDP is adopted to measure R&D level. The increase in R&D spending can enhance energy efficiency and energy transition, which is critically important for curbing energy use-related carbon emissions [41].

#### 4.2.4. Mediating Variables

As discussed above, this study shows that digital economy may indirectly affect the *CEI* by optimizing the energy consumption structure, improving the total factor energy productivity, and promoting green technology innovation. Therefore, three mediating variables are further selected to perform mechanism analyses:

Energy consumption structure (*ECS*): Coal plays a dominant role in China’s primary energy composition in comparison to natural gas, electric power, oil, and other energy sources. It accounts for approximately 70% of the main energy consumption and 80% of electricity generation in China [14,92]. Accordingly, the indicator of ECS can be determined by the proportion of coal consumption in aggregate energy consumption.

Total factor energy productivity (*TFEP*): In order to achieve environmental sustainability by conserving energy and reducing pollution emissions, the TFEP has been proposed as a method of evaluating regional energy efficiency. It is defined as an indicator of energy input–output derived from the theory of total factor productivity. Following the practice of Hu and Wang [93] and Tang et al. [94], this study utilizes the DEA–Malmquist index method to measure the TFEP. The basic variables include the input indicator (capital stock, labor employment, and energy consumption) and the output indicator (regional GDP).

Green technology innovation (*GTI*): Green technologies are defined as environmentally friendly technologies that protect the ecological environment. In research, patents are often used as an indicator of technological innovation. Based on the study by Xu et al. [95], Lee et al. [96], and Li et al. [97], this paper applies the number of green invention patent applications as the indicator of regional GTI. By analyzing the green patents classification provided by the World Intellectual Property Organization (WIPO) and the Chinese patent data, it can obtain total green patent applications at different province levels in China.

### 4.3. Data Source and Description

Since some indicators of the digital economy are largely unavailable before 2011, this study selects the panel data of 30 provinces from 2011 to 2019 for empirical analysis, excluding Tibet, Taiwan, Macao, and Hong Kong due to the availability of related indicators. The data of the above variables are collected from the China Statistical Yearbook, the China Energy Statistical Yearbook, the China Environmental Statistical Yearbook, the China Labor Statistical Yearbook, and Statistical Yearbook of each province. Table 4 provides the definitions and descriptive statistical results of the specific indicators.

As shown in Table 4, the logarithm value of the CEI ranges from −1.613 to 2.553, with an average of 0.511, showing that carbon dioxide emissions vary greatly among different provinces. Meanwhile, the largest logarithm value of the digital economy index is 4.302 and its smallest value is 0.788, illustrating that regional variations are also evident in the digital economy development. As for the control variables, the average logarithm of economic development, urbanization, FDI, Trade, and R&D are 9.724, 4.034, 0.352, 2.889, and 0.363, respectively. This implies that there are significant differences among regional macroeconomic characteristics, and that they should be controlled due to their impact on carbon dioxide emissions.

## 5. Results and Discussion

**Figure 2:**
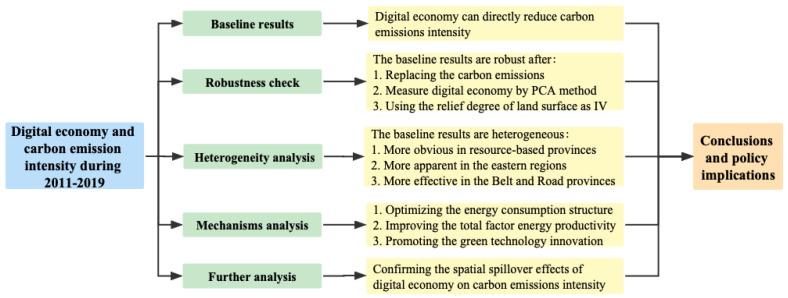
The main findings of the effect of digital economy on carbon emission intensity.

This section examines the impact of digital economy on an environmental sustainability proxy by carbon emission intensity. The endogenous issue is then addressed based on the instrument variable method. Moreover, a series of heterogeneity analysis are reported and transmission mechanisms are discussed through the mediating effect model. Finally, the spatial spillover effects of digital economy on carbon emission intensity are also provided. To show the findings of the study in a clear manner, we have drawn the following Figure 2 to summarize and present them.

### 5.1. Baseline Regression Results

The study estimates the equation (1) using the ordinary least squares (OLS) regression for evaluating the direct effect of digital economy on environmental sustainability. Table 5 presents the estimation results of the benchmark model; Column (1) includes no control variables, whereas control variables and the two-way fixed effects are added sequentially to Columns (2) and (3), respectively. As shown in Table 5, all the DEI coefficients are negative and significant at the 1% level, illustrating that digital economy can directly reduce carbon emission intensity. In particular, the DEI is significantly negative at the 1% level with an estimated coefficient of −0.324 in Column (3), implying that a 1% increase in the DEI leads to a 3.24% decrease in CEI, which indicates that digital economy can pave the way towards environmental sustainability. The results are consistent with Hypothesis 1 but the conclusion is contrary to the findings of Asongu et al. [5] and Weili et al. [51], who found that ICT technology increases carbon emissions in sub-Saharan Africa and the Belt and Road countries. The possible reason lies in the different research objects. African and most of the Belt and Road countries have a less developed digital economy than China, and increasing ICT usage results in higher energy consumption and carbon emissions in these countries.

In addition, the results of control variables in Column (3) are in line with prior studies. Specifically, a significant negative relationship exists between economic development and CEI, which supports the “environmental Kuznets curves” hypothesis in China. Urbanization is significantly positive with CEI, which is consistent with the study of Zi et al. [28], who emphasize the carbon emissions driven by China’s rapid urbanization. Trade structure has a significantly negative correlation with CEI that is consistent with the pollution heaven theory. In contrast to expectations, the research finds no significant links between R&D level and CEI, implying that it is important to explore how to achieve environmental sustainability through R&D in China.

### 5.2. Robustness Check

#### 5.2.1. Substitution Variable Method

To check the robustness of the baseline regression results, this study further employs the substitution variable method to verify its reliability. This paper uses other indicators to replace the dependent variable. Specifically, referring to Li et al. [98], we adopt total carbon dioxide emissions (CE) and per capita carbon dioxide emissions (CEP) as the proxy variables of CEI, and the results are shown in Columns (1) and (2) of Table 5, respectively. The regression coefficients of DEI are all negative and significant at the 5% level, indicating a significant negative correlation persists between DEI and carbon emissions after using different carbon dioxide emissions indicators. In terms of control variables, the estimation results are in line with the baseline regression results. It further confirms the reliability of the Hypothesis 1 that a digital economy can directly suppress carbon dioxide emissions in China.

#### 5.2.2. Different Setting for Digital Economy

Compared with the improved entropy weight method, existing studies also employ the principal component analysis (PCA) method to construct a digital economy index. Referring to Pan et al. [73] and Wang et al. [99], the core explanatory variable digital economy has also been calculated by PCA method, and the results are presented in Column (3) of Table 5. It can be seen from Column (3) that there is still a significant negative relationship between DEI and CEI after considering the two different settings for constructing a digital economy index. As a result, the robustness of the benchmark regression results has been further demonstrated.

#### 5.2.3. The Treatment of Endogeneity: Instrumental Variable

Potential endogeneity issues may affect the reliability of the baseline regression results. For instance, it is more conducive to digital economy development if a province has higher carbon emission intensity and digital infrastructure construction requirements. As a result, a reverse causality problem is inherent in the baseline model. Moreover, there may still be unobservable factors affecting carbon emission intensity due to the availability of data. Therefore, this research attempts to alleviate the endogenous problem by applying the instrumental variable method.

Referring to Nunn and Qian [100] and Luo et al. [60], this study constructs the interaction term between the relief degree of land surface in the province (related to individual) and the number of post offices per 10,000 people (related to time) as an instrumental variable. Post offices, as a central facility, have been important in connecting information communication and developing economies. In addition, the terrain’s ups and downs have no direct relationship with the carbon emission intensity. The instrumental variable regression results are reported in Column (4) of Table 6. It can be seen from the KP-LM statistics that there is no insufficient identification of instrumental variables. The results of KP-Wald F statistics are higher than the critical value of 16.38 at the 10% level, indicating that there is no weak instrumental variable problem. The regression results in Column (4) reveal that digital economy development can significantly inhibit carbon emission intensity. By solving the possible endogenous problems using instrumental variable estimation, digital economy can still help pave the way towards environmental sustainability; this confirms the robustness of the baseline regression results.

### 5.3. Heterogeneity Analysis

According to the above regression results, digital economy development is beneficial for reducing China’s carbon emission intensity; however, this effect may be regionally heterogeneous. Accordingly, this paper classifies China into different regions to investigate the heterogeneous environmental effects of the digital economy.

#### 5.3.1. Regional Heterogeneity in Resource Endowment

Resource-based provinces tend to attract more energy-intensive industries and increase carbon emissions. Thus, it is essential to separate the sample provinces into resource-based and non-resource-based to investigate the heterogeneous effect of digital economy on carbon emission intensity. Following the study of Li et al. [98] and a comprehensive analysis of the output value and employment proportion of resource-based industries, nine provinces are classified as resource-based provinces, including Shanxi, Inner Mongolia, Guizhou, Shaanxi, Xinjiang, Yunnan, Ningxia, Heilongjiang, and Qinghai. The regression results in Columns (1) and (2) of Table 7 show that digital economy reduces carbon emission intensity in resource-based provinces but not in non-resource-based provinces. The main reason is that resource-based provinces have ample mineral, coal, and fossil fuel resources, and they tend to result in more energy consumption and carbon emissions. Digital economy development can contribute to the improvement of resource mining technology and reduce energy consumption. Consequently, there is a more obvious dividend effect of digital economy in resource-based provinces.

#### 5.3.2. Regional Heterogeneity in Economic Development

From the perspective of economic development, China’s economy has significant spatial differences. A noticeable development gap can be observed among the eastern, central, and western regions of China. Thus, this study further divides the 30 sample provinces into three major economic regions: the eastern region comprises the 11 provinces of Hebei, Beijing, Tianjin, Fujian, Shandong, Jiangsu, Zhejiang, Shanghai, Liaoning, Hainan, and Guangdong; the central region consists of 8 provinces including Hunan, Anhui, Jilin, Henan, Heilongjiang, Hubei, Jiangxi, and Shanxi; the final 11 sample provinces make up the western region, including Guangxi, Shaanxi, Gansu, Chongqing, Sichuan, Inner Mongolia, Xinjiang, Ningxia, Guizhou, Qinghai, and Yunnan. As shown in Columns (3) to (5) of Table 7, the estimated coefficients of DEI are only significantly negative at the 1% level in the eastern regions, suggesting that the carbon reduction effect of the digital economy is more apparent in the eastern regions; this is probably because of the higher digital economy development level in the eastern regions than in the central and western regions, which contributes to a reduction in carbon dioxide emissions.

#### 5.3.3. Regional Heterogeneity in Openness

Nowadays, China is proposing the “One Belt and One Road” project to facilitate regional integration, extend global value chains, and improve energy resources allocation efficiency. There are big differences among provinces along the Belt and Road in technical development and energy consumption, resulting in the heterogeneity of carbon emissions. Therefore, this study further breaks the 30 sample provinces down based on whether they are the Belt and Road provinces or not. Following the classification of Li et al. [101], 17 provinces along China’s Belt and Road (except Tibet) are collected, including Inner Mongolia, Fujian, Guangxi, Ningxia, Chongqing, Shanghai, Heilongjiang, Xinjiang, Liaoning, Guangdong, Jilin, Yunnan, Gansu, Zhejiang, Shaanxi, Hainan, and Qinghai. The estimated results are shown in Columns (6)–(7) of Table 7. The results show that the coefficient of digital economy is significantly negative in the Belt and Road provinces, while it is negative but not significant in the Non-Belt and Road provinces. The main explanation for the difference is that the Belt and Road strategy has promoted the reform and openness in the provinces along the route, leading to better improvement of network infrastructure and technological development. Thus, the inhibiting effect of digital economy on carbon emission intensity is more effective.

### 5.4. Transmission Mechanisms Analysis

The above research results reveal that digital economy can directly suppress China’s carbon emission intensity and promote environmental sustainability. This study further explores the transmission mechanisms through which digital economy indirectly affects China’s carbon emission intensity. According to hypothesis 2 and the mediating effect model, the above Equations (2) and (3) are estimated to test the mechanisms. Specifically, based on the theoretical analysis, this research considers the transmission mechanisms from three perspectives: energy consumption structure (ECS), total factor energy productivity (TFEP), and green technology innovation (GTI). The results are reported in Table 8.

#### 5.4.1. Mechanism I: Optimizing the Energy Consumption Structure

In Table 8, Column (1) explores the impact of digital economy on energy consumption structure, and this impact is significantly negative at the 1% level. The results in Column (1) unveil that digital economy can significantly decrease the proportion of coal consumption in aggregate energy consumption. The results in Column (2) show that the DEI is still significantly negative at the 1% level after adding the ECS, and the estimated coefficient of the DEI changes from −0.324 in the baseline regression to −0.298. The results support mechanism I, confirming that digital economy can indirectly inhibit carbon emission intensity through optimizing the energy consumption structure. This result is in line with the prior study of Ren et al. [102], which emphasizes Internet development to optimize the energy consumption structure through an industrial structural upgrading effect.

#### 5.4.2. Mechanism II: Improving the Total Factor Energy Productivity

Columns (3) and (4) in Table 8 provides the estimation results of how digital economy indirectly inhibits carbon emission intensity by improving the total factor energy productivity. First, results in Column (3) indicate that the digital economy has a significant positive impact on the TFEP, the TFEP increases by 10% when the digital economy rises by 1%. Second, the estimated coefficients of DEI and TFEP in Column (4) are all significantly negatively correlated with CEI. In particular, compared to the baseline regression, the coefficient of DEI drops from −0.324 to −0.265, a decrease of 18.2%. Consequently, the results support mechanism II, proving that digital economy can promote environmental sustainability by improving the TFEP. This result confirms the factor effect of digital economy on carbon emission intensity.

#### 5.4.3. Mechanism III: Promoting the Green Technology Innovation

As shown in Table 8, Column (5) gives the estimation results of the effect of digital economy on GTI, whereas the estimation results regarding the mediation effect of GTI are reported in Column (6). The results in Column (2) reveals that digital economy is significantly and positively correlated with GTI, indicating that digital economy can improve China’s green technology innovation. This result is consistent with the study of Luo et al. [60]. In addition, the results of Column (2) suggest that digital economy and green technology innovation are all significantly and negatively related to carbon emission intensity. Therefore, enhancing the carbon emissions reduction effect of digital economy through green technology innovation is conducive to achieving China’s environmental sustainability. The results confirm the technical effect of digital economy and support Mechanism III.

### 5.5. Further Analysis: Spatial Spillover Effect

Due to the continuous increase in economic interconnection between regions, it is gradually becoming more apparent that there are some interaction effects between these regions. The carbon dioxide emissions of one province may be affected by the other provinces, resulting in spatial autocorrelation. At the same time, the digital economy can break through the limitation of time and space and help realize a cross-regional division of labor and cooperation. It appears that digital economy in one region can also affect carbon dioxide emissions of other regions. Therefore, this paper further discusses the relationship between digital economy and environmental sustainability using a spatial econometric method.

#### 5.5.1. Spatial Correlation Test

This study first utilizes the Moran’s I index to examine whether a spatial autocorrelation exists in carbon emission intensity under three different spatial weighted matrices, namely contiguity-based, economic-based, and distance-based spatial weighted matrices. It can be seen in Table 9 that the Moran’s index values of carbon emission intensity from 2011–2019 are significantly positive at the 1% and 5% levels, thereby the null hypothesis of no spatial autocorrelation of carbon emission intensity is significantly rejected. Therefore, in the case of China’s carbon emissions, there is a significant spatial autocorrelation, which makes it appropriate to use spatial econometric analysis.

#### 5.5.2. Spatial Spillover Effect Test

This paper focuses on examining the spatial spillover effect of digital economy on carbon emission intensity by utilizing the spatial Durbin model. In view of this, this study takes the practice of Elhorst [84] as reference to decompose the impact of digital economy on carbon emission intensity, including the direct effect, indirect effect and overall effect. Specifically, the direct effect represents the impact of local digital economy on regional carbon emission intensity. The indirect effect, also known as spatial spillover effect refers to the impact of digital economy in neighboring regions on local carbon emission intensity. The total effect is the sum of direct effect and indirect effect. The estimated results of the spatial Durbin model are listed in Table 10.

As shown in Table 10, the coefficients of digital economy and its spatial interaction term are all significantly negative, indicating that the sample provinces not only have exogenous digital economy interaction effects, but also have endogenous interaction effects of carbon emission intensity. Moreover, according to the decomposition effects, the direct, indirect, and overall effects of digital economy on carbon emission intensity are also all significantly negative at the 1% and 5% level. These results show that digital economy will not only inhibit the carbon emission intensity of the local area, but also help to reduce the carbon emission intensity of neighboring areas, thus digital economy development has spatial spillover effects. Therefore, the argument in Hypothesis 3 is supported. The conclusion coincides with those of Luo et al. [60] and Lin et al. [47], who find that there is also a spatial spillover effect between digital economy and green innovation, as well as between Internet development and carbon emission performance.

## 6. Conclusions and Policy Implications

### 6.1. Conclusions

This study utilizes the panel data of 30 provinces in China from 2011 to 2019 to empirically investigate the effect of digital economy on an environmental sustainability proxy by reducing carbon emission intensity. It first constructs a digital economy indicator by applying the improved entropy weight method from the digital production and digital application sector, and then uses the fixed effects model, mediation effect model, and spatial panel Durbin model to accurately grasp the relationship between digital economy and environmental sustainability. The main conclusions are summarized as follows:

First, digital economy has a significant negative effect on carbon emission intensity. A 1% increase in digital economy development can lead to a 3.24% decrease in carbon emission intensity, illustrating that digital economy can pave the way towards environmental sustainability in China. The conclusion remains robust after conducting several robustness checks, such as changing the dependent variable, different setting for digital economy, and endogenous treatment with an instrumental variable.

Second, the effect of digital economy on carbon emission intensity has significant regional heterogeneity. Digital economy significantly reduces carbon emission intensity in resource-based provinces, as well as the eastern regions and the Belt and Road provinces that are more economically developed and open. In contrast, the effect is not obvious in the non-resource-based provinces, central and western regions, and the Non-Belt and Road provinces.

Third, the analysis of transmission mechanisms of digital economy on environmental sustainability reveals that digital economy can indirectly inhibit carbon emission intensity through optimizing the energy consumption structure, improving the total factor energy productivity and promoting the green technology innovation. In addition, the spatial effect analysis shows that digital economy can not only boost the environmental sustainability of the local area, but also of the neighboring areas, confirming the spatial spillover effects of digital economy development.

### 6.2. Policy Implications

The above findings provide significant policy implications for developing countries to achieve environmental sustainability.

First, policies aimed at strengthening the construction of digital facilities should be encouraged in developing countries. The government should integrate local advantages and accelerate the commercialization of 5G, absorb foreign advanced digital technologies and ideas, and further release the dividends of digital economy’s carbon reduction effect.

Second, in less developed areas, digital technology and digital knowledge need to be widely popularized due to the unbalanced digital economy development. The government should encourage cooperation between regions and strengthen assistance and cooperation among provinces with different development levels.

Third, the government should promote the digitalization of energy industries to create new energy models and improve energy efficiency, as well as stimulate green environmental technology innovation. Moreover, local governments should set up a platform to facilitate the exchange of regional knowledge communication.

## Figures and Tables

**Table 1:** Studies on the relationship between digital economy and environmental sustainability.

Literature	Data	Methods	Key Findings
(a) Single digital economy-related indicator			
Salahuddin et al. [46]	1991–2012; OECD countries	PMG	Internet usage→(+) CO_2_ emissions
Lin and Zhou [47]	2006–2017; Chinese provinces	FE, Entropy weight method	Internet usage→(+) energy and carbon emissions performance
Wang et al. [48]	2006–2017; Chinese provinces	GMM, IV-GMM	Internet economy→(+) carbon emissions efficiency
Liang et al. [49]	2001–2017; Chinese cities	FMOLS, DOLS	E-commerce→ carbon emissions: an inverted-U relationship; carbon Kuznets curve (CKC)
Ozturk and Ullah [24]	2007–2019; OBRI countries	OLS, 2SLS, GMM	Digital finance→(+) carbon emissions
Usman et al. [50]	1990–2018; 9 Asian countries	OLS, FE, GLS	ICT→(+) carbon emissions
Weili et al. [51]	2000–2019; Belt and Road countries	OLS, FE, GLS	ICT→(+) carbon emissions
(b) Direct effect of digital economy on environmental quality indicators			
Asongu et al. [5]	2000–2012; Sub-Saharan Africa	GMM	ICT penetration→(+) carbon emissions;amimiMobile phone→(−) carbon emissions
Jiang et al. [11]	2016.01–2018.06; Chinese cities and industrial sectors	System dynamics amimisimulation	Bitcoin blockchain→(+) carbon emissions
Chiabai et al. [7]	2009.11–2010.01; Chinese cities and industrial sectors	Questionnaire survey	ICT tools→ environmental sustainability: consider user preferences
Bastida et al. [8]	2015; EU household sector	Quantitative analysis	ICT→(−) greenhouse gases
Chen [52]	1990–2018; BRICS countries	ARDL	Digitization→(−) CO_2_ emissions
Amri et al. [53]	1975–2014; Tunisia	ARDL	ICT→CO_2_ emissions: no significant impact
N’dri et al. [54]	1990–2014; 58 developing countries	PMG-ARDL	ICT→(−)CO_2_ emissions: low-income developing countries amimiICT ≠ CO_2_ emissions: high-income developing countries
(c) Indirect effect of digital economy on environmental development			
Zhang et al. [55]	2012–2019; Chinese enterprises	FE, DID	Digital economy→(+) corporate energy conservation and emission reduction
Shahbaz et al. [56]	2013–2019; 72 countries	SYS-GMM	Digital economy→(+) energy transition
Xu et al. [57]	2000–2019; 109 countries	SYS-GMM	Digitalization→(+) energy structure
Canzian et al. [58]	2008–2014; 1209 companies	FE, DID	Broadband upgrade→(+) TFP
Liu et al. [59]	2011–2019; Chinese 286 cities	Tobit, PVAR	Digitalization→(+) GTFP
Luo et al. [60]	2011–2019; Chinese 286 cities	OLS, PCA, Spatial Durbin model, DID	Digital economy→(+) Urban green innovation
Tian et al. [61]	2012–2018; Chinese provinces	OLS, threshold model	Digital economy→(+) green financial investment

Note: (1) Abbreviations used for the data are as follows: OECD: Organization for Economic Cooperation and Development; OBRI: One Belt and Road Initiative; EU: European Union; BRICS: Brazil, Russia, India, China, South Korea. (2) Abbreviations used for the methods are as follows: PMG: Pooled Mean Group; FE: Fixed Effect; GMM: Generalized Method of Moments; IV-GMM: Instrumental Variable Generalized Method of Moments; FMOLS: Fully-Modified Ordinary Least Squares; DOLS: Dynamic Ordinary Least Squares; OLS: Ordinary Least Squares; 2SLS: Two-Stage Least Squares; GLS: Generalized Least Squares; ARDL: Autoregressive Distributed Lag; DID: Differences-in-Differences; SYS-GMM: System Generalized Method of Moments; PVAR: Panel Vector Autoregressive; PCA: Principal Component Analysis. (3) Abbreviations used for the key findings are as follows: → implies a relationship; →(+) means a positive relationship; →(−) indicates a negative relationship; TFP: Total Factor Productivity; PCA: Green Total Factor Productivity.

**Table 2:** The carbon emissions parameters of major energy sources.

	Natural Gas	Kerosene	Gasoline	Coke	Diesel	Coal	Fuel Oil
*CAC* (unit: tC/tJ)	15.32	19.60	18.90	29.41	20.17	27.28	21.09
*CAV* (unit: kJ/kg)	38,931	44,750	44,800	28,435	43,330	17,824	40,190
*COF*	0.99	0.99	0.98	0.93	0.98	0.92	0.99

Note: *CAC* represents the carbon content of various energy, *CAV* refers to the average calorific value, and *COF* denotes the carbon oxidation factor.

**Table 3:** The indicator system of measuring the digital economy.

Criterion Layer	Index Layer	Unit	Direction	Weight
Digital industrialization	Total telecommunications revenue per capita	yuan	+	0.099
	Software revenue per capita	yuan	+	0.105
	Ratio of information industry practitioners	%	+	0.151
Digital transaction	Digital financial inclusion index	-	+	0.024
	Express delivery revenue per capita	yuan	+	0.181
	Added value of tertiary industry/GDP	%	+	0.023
Digital infrastructure	Broadband Internet penetration	%	+	0.148
	Mobile phone penetration	%	+	0.085
	Long-distance optical cable density	km	+	0.091
	Number of domain names per thousand people	unit	+	0.023
	Internet access port density	unit	+	0.035
Digital literacy	Average years of education	year	+	0.021
	Education expenditure/Total fiscal expenditure	%	+	0.014

Note: + indicates the positive effect of the indicator on digital economy.

**Table 4:** Descriptive statistics of the variables.

Variables	Definition	Obs	Mean	SD	Min	Max
Dependent Variable						
*lnCEI*	Carbon emission intensity	270	0.511	0.815	−1.613	2.553
Core Explanatory Variable						
*lnDEI*	Digital economy index	270	2.351	0.625	0.788	4.302
Control Variables						
*lnRgdp*	Economic development	270	9.724	0.872	7.223	11.463
*lnUrban*	Urbanization level	270	4.034	0.203	3.537	4.545
*lnFDI*	Foreign direct investment	270	0.352	1.088	−3.293	2.534
*lnTrade*	Trade structure	270	2.889	0.928	0.243	5.009
*lnRdl*	Research and development level	270	0.363	0.582	−0.862	1.841
Mediating Variables						
*lnECS*	Energy consumption structure	270	−0.195	0.570	−3.695	0.901
*lnTFEP*	Total factor energy productivity	270	−0.751	0.327	−1.457	0.131
*lnGTI*	Green technology innovation	270	7.432	1.397	2.708	10.354

**Table 5:** The effect of digital economy on carbon emission intensity.

Variables	Dependent Variable: *lnCEI*		
	(1)	(2)	(3)
*lnDEI*	−0.630 ***	−0.740 ***	−0.324 ***
	(0.066)	(0.091)	(0.095)
*lnRgdp*		−0.140 ***	−1.724 ***
		(0.053)	(0.279)
*lnUrban*		2.360 ***	1.028 ***
		(0.436)	(0.268)
*lnFDI*		−0.165 ***	−0.005
		(0.044)	(0.020)
*lnTrade*		−0.324 ***	0.127 ***
		(0.065)	(0.040)
*lnRdl*		−0.341 ***	0.144
		(0.105)	(0.097)
Year fixed effects	No	No	Yes
Province fixed effects	No	No	Yes
Observations	270	270	270
R^2^	0.233	0.538	0.982

Note: *** indicates 1% level of statistical significance. Robust standard errors in parentheses, the same as below.

**Table 6:** Robustness test of the effect of digital economy on environmental sustainability.

Variables	Dependent Variable			
	(1) *lnCE*	(2) *lnCEP*	(3) *lnCEI*	(4) *lnCEI*
*lnDEI*	−0.239 **	−0.204 **	−0.114 **	−0.348 ***
	(0.093)	(0.090)	(0.045)	(0.058)
*lnRgdp*	0.072	−0.355	0.096	−1.716 ***
	(0.273)	(0.267)	(0.271)	(0.329)
*lnUrban*	0.475 *	0.830 ***	−0.305	1.045 **
	(0.262)	(0.256)	(0.338)	(0.457)
*lnFDI*	−0.017	−0.011	−0.008	−0.004
	(0.020)	(0.019)	(0.020)	(0.021)
*lnTrade*	0.117 ***	0.116 ***	0.081 **	0.129 **
	(0.039)	(0.038)	(0.041)	(0.052)
*lnRdl*	0.069	0.026	0.014	0.152
	(0.095)	(0.093)	(0.095)	(0.195)
Year fixed effects	Yes	Yes	Yes	Yes
Province fixed effects	Yes	Yes	Yes	Yes
KP-LM statistics				9.352 ***
				[0.001]
KP-Wald F statistics				38.109
				[16.38]
Observations	270	270	270	270
R^2^	0.981	0.975	0.981	0.245

Note: *, **, and *** indicate 10%, 5%, and 1% levels of statistical significance, respectively. The value in [ ] of KP-LM statistics is the *p*-value of the coefficient. The value in [ ] of KP-Wald F statistics represents the critical value of the weak instrumental variable Stock–Yogo test at the 10% level.

**Table 7:** Heterogeneous effect of digital economy on carbon emission intensity.

Variables	Resource	Non-Resource	Eastern	Central	Western	Belt	Non-Belt
	(1)	(2)	(3)	(4)	(5)	(6)	(7)
*lnDEI*	−0.856 ***	−0.030	−0.713 ***	0.117	0.049	−0.420 ***	−0.220
	(0.219)	(0.102)	(0.143)	(0.370)	(0.121)	(0.106)	(0.178)
*lnRgdp*	−2.770 ***	−1.000 ***	−2.484 ***	−4.372 ***	−0.065	−1.167 ***	−2.552 ***
	(0.517)	(0.332)	(0.509)	(1.149)	(0.394)	(0.322)	(0.508)
*lnUrban*	0.327	0.687 ***	−0.226	2.773 *	1.026 ***	0.709 **	2.527 ***
	(1.040)	(0.238)	(0.672)	(1.520)	(0.301)	(0.308)	(0.574)
*lnFDI*	0.046	−0.065 ***	0.058 **	−0.161	−0.102 ***	0.034	−0.027
	(0.033)	(0.023)	(0.027)	(0.110)	(0.030)	(0.021)	(0.050)
*lnTrade*	0.180 ***	−0.045	0.127 **	−0.044	0.227 *	0.139 ***	0.015
	(0.060)	(0.051)	(0.051)	(0.128)	(0.121)	(0.042)	(0.091)
*lnRdl*	0.687 ***	0.006	0.463 ***	0.767 **	−0.103	0.207 *	−0.091
	(0.187)	(0.107)	(0.138)	(0.330)	(0.138)	(0.109)	(0.193)
Year fixed effects	Yes	Yes	Yes	Yes	Yes	Yes	Yes
Province fixed effects	Yes	Yes	Yes	Yes	Yes	Yes	Yes
Observations	81	189	99	72	99	153	117
R^2^	0.970	0.980	0.988	0.983	0.984	0.986	0.982

Note: *, **, and *** indicate 10%, 5%, and 1% levels of statistical significance, respectively.

**Table 8:** Mechanism test of the effect of digital economy on carbon emission intensity.

Variables	Dependent Variable					
	*lnECS*	*lnCEI*	*lnTFEP*	*lnCEI*	*lnGTI*	*lnCEI*
	(1)	(2)	(3)	(4)	(5)	(6)
*lnDEI*	−0.221 ***	−0.298 ***	0.100 **	−0.265 ***	0.458 ***	−0.266 ***
	(0.061)	(0.087)	(0.040)	(0.092)	(0.173)	(0.097)
*lnECS*		0.313 ***				
		(0.042)				
*lnTFEP*				−0.462 ***		
				(0.158)		
*lnGTI*						−0.101 ***
						(0.037)
*lnRgdp*	−0.666 ***	−1.171 ***	0.217 *	−1.407 ***	0.996 *	−1.407 ***
	(0.179)	(0.259)	(0.118)	(0.282)	(0.508)	(0.283)
*lnUrban*	0.758 ***	0.123	−0.415 ***	0.715 ***	0.445	0.951 ***
	(0.170)	(0.264)	(0.112)	(0.274)	(0.483)	(0.267)
*lnFDI*	0.014	0.026	0.016 *	0.019	−0.018	0.010
	(0.013)	(0.019)	(0.009)	(0.021)	(0.037)	(0.020)
*lnTrade*	0.051 *	0.092 **	0.061 ***	0.147 ***	−0.004	0.118 ***
	(0.027)	(0.038)	(0.018)	(0.043)	(0.076)	(0.042)
*lnRdl*	−0.075	0.124	−0.010	0.145	−0.043	0.146
	(0.064)	(0.092)	(0.043)	(0.101)	(0.183)	(0.101)
Year fixed effects	Yes	Yes	Yes	Yes	Yes	Yes
Province fixed effects	Yes	Yes	Yes	Yes	Yes	Yes
Observations	270	270	270	270	270	270
R^2^	0.976	0.985	0.980	0.982	0.980	0.982

Note: *, **, and *** indicate 10%, 5%, and 1% levels of statistical significance, respectively.

**Table 9:** The spatial correlation test results.

Year	Spatial Weights Matrix Type					
	Contiguity-Based		Economic-Based		Distance-Based	
	Moran’s I	Z Value	Moran’s I	Z Value	Moran’s I	Z Value
2011	0.476 ***	4.152	0.315 **	2.394	0.098 ***	2.897
2012	0.479 ***	4.170	0.349 ***	2.619	0.101 ***	2.941
2013	0.433 ***	3.827	0.348 ***	2.635	0.091 ***	2.749
2014	0.457 ***	4.009	0.395 ***	2.950	0.094 ***	2.818
2015	0.464 ***	4.066	0.379 ***	2.838	0.089 ***	2.690
2016	0.421 ***	3.726	0.352 ***	2.660	0.079 **	2.495
2017	0.421 ***	3.723	0.352 ***	2.657	0.076 **	2.413
2018	0.424 ***	3.755	0.335 **	2.550	0.079 **	2.481
2019	0.429 ***	3.795	0.331 **	2.515	0.076 **	2.428

Note: **, and *** indicate 5%, and 1% levels of statistical significance, respectively.

**Table 10:** The spatial regression results under three spatial weighted matrices.

Variables	Dependent Variable: *lnCEI*		
	Contiguity-Based	Economic-Based	Distance-Based
	(1)	(2)	(3)
*lnDEI*	−0.392 ***	−0.364 ***	−0.305 ***
	(0.084)	(0.083)	(0.084)
W * lnDEI	−0.156 **	−0.121 ***	−0.293 **
	(0.081)	(0.045)	(0.140)
*lnRgdp*	−1.792 ***	−1.975 ***	−1.685 ***
	(0.332)	(0.334)	(0.297)
*lnUrban*	1.233 *	1.127	1.388 **
	(0.654)	(0.686)	(0.627)
*lnFDI*	0.603	0.190	0.471
	(0.755)	(0.839)	(0.754)
*lnTrade*	0.324 **	0.473 ***	0.287 **
	(0.150)	(0.143)	(0.142)
*lnRdl*	0.744	6.054	−2.421
	(5.025)	(5.285)	(4.990)
Year fixed effects	Yes	Yes	Yes
Province fixed effects	Yes	Yes	Yes
Direct effect	−0.391 ***	−0.369 ***	−0.313 ***
	(0.084)	(0.087)	(0.084)
Indirect effect	−0.134 ***	−0.152 **	−0.343 **
	(0.053)	(0.077)	(0.150)
Overall effect	−0.525 **	−0.521 **	−0.656 ***
	(0.182)	(0.112)	(0.155)
Observations	270	270	270
R^2^	0.240	0.252	0.176

Note: *, **, and *** indicate 10%, 5%, and 1% levels of statistical significance, respectively.

## Data Availability

The data involved in this study are all from public data.
